# Lead Concentration in Long-Tailed Macaque (*Macaca fascicularis*) Hair in Kuala Selangor, Malaysia

**DOI:** 10.21315/tlsr2018.29.2.12

**Published:** 2018-07-06

**Authors:** Nurul Ashikin Hassim, Kamarul Hambali, Nor Shahirul Umirah Idris, Aainaa Amir, Ahmad Ismail, Syaizwan Zahmir Zulkifli, Ai Yin Sow

**Affiliations:** 1 Faculty of Earth Science, Universiti Malaysia Kelantan, Locked Bag No. 100, Jeli Campus, 17600 Jeli, Kelantan, Malaysia; 2 Department of Biology, Faculty of Science, Universiti Putra Malaysia, 43400 UPM Serdang, Selangor, Malaysia

**Keywords:** Lead, Long-tailed Macaque, *Macaca fascicularis*, Ecotoxicology, Kuala Selangor, Plumbum, Kera Ekor Panjang, *Macaca fascicularis*, Ekotoksikologi, Kuala Selangor

## Abstract

Long-tailed macaque (*Macaca fascicularis*) has the potential to be a good biological indicator for toxic exposure because they have an almost similar physiology and behaviour to humans. The objective of this study is to determine the concentration of lead (Pb) in hair samples of long-tailed macaques which were found in and out of the Kuala Selangor Nature Park (KSNP) area. The hypothesis is long-tailed macaques that live in the anthropogenic area (outside KSNP) may be exposed to high levels of lead compared to long-tailed macaques living in the forest area (inside KSNP). Analysis of hair samples were carried out using Inductively Coupled Plasma-Mass Spectrometry (ICP-MS). The study found that the average mean of lead concentration in the anthropogenic area is 6.31 μg/g while for the forest area it is 3.16 μg/g. Lead concentration in the two areas are statistically insignificant. Nevertheless, lead concentration in the anthropogenic area recorded a slightly higher mean concentration than in the forest area. Even so, results of this study indicate that long-tailed macaques in Kuala Selangor are not exposed to high levels of lead. This study is the first in Malaysia to utilise long-tailed macaques as a biological indicator for testing the concentration of toxic substances in the environment. This study is still in its early stages; thus, future research requires improvements.

## Abstract (ms)

Kera ekor panjang (*Macaca fascicularis*) mempunyai potensi untuk menjadi penunjuk biologi yang baik untuk pendedahan toksik kerana mempunyai fisiologi dan tingkah laku yang hampir sama dengan manusia. Objektif kajian ini adalah untuk menentukan kepekatan plumbum (Pb) dalam sampel rambut kera ekor panjang yang dijumpai di dalam dan luar dari kawasan Taman Alam Kuala Selangor (KSNP). Secara hipotesisnya, kera ekor panjang yang tinggal di kawasan antropogenik (di luar KSNP) mungkin terdedah kepada paras plumbum yang tinggi berbanding dengan kera ekor panjang yang tinggal di kawasan hutan (di dalam KSNP). Analisis sampel rambut dijalankan dengan menggunakan mikroplasma gandingan teraruh-jirim spektrometri (ICP-MS). Kajian mendapati bahawa purata kepekatan plumbum di kawasan antropogenik adalah 6.31 μg/g manakala bagi kawasan hutan adalah 3.16 μg/g. Kepekatan plumbum di kedua-dua kawasan secara statistik tidak begitu ketara. Walaubagaimanapun, kepekatan plumbum di kawasan antropogenik mencatat kepekatan purata sedikit lebih tinggi daripada di kawasan hutan. Walaupun begitu, hasil kajian ini menunjukkan bahawa kera ekor panjang di Kuala Selangor tidak terdedah kepada tahap plumbum yang tinggi. Kajian ini adalah yang pertama di Malaysia menggunakan kera ekor panjang sebagai penunjuk biologi untuk menguji kepekatan bahan toksik di alam sekitar. Kajian ini masih dalam peringkat awal; oleh itu, penyelidikan masa depan memerlukan penambahbaikan.

## INTRODUCTION

Heavy metals play an important role in various oxidation-reduction reactions and are important constituents of several key enzymes (World Health Organization 1996). Nevertheless, a large amount of heavy metals can lead to poisoning and cause chronic toxicity. Unlike many organic pollutants, which eventually degrade to carbon dioxide and water, heavy metals tend to accumulate in the environment (Wang 2009). Many heavy metals are essential in small quantities for human health. Eventually, they become concentrated as a result of human caused activities. Common sources are mining and industrial wastes, vehicle emissions, lead acid batteries, fertilisers, paints, treated woods and aging water supply infrastructure (Harvey *et al.* 2015). The most common heavy metal pollutants are arsenic (As), cadmium (Cd), chromium (Cr), lead (Pb), and mercury (Hg). Commonly, human and animals may be exposed to the toxicity via routes such as contamination of drinking water, food, soil, medicines, improperly coated food containers, ingestion of lead-based paints, air or water pollution, dust, and industrial activities.

Long-tailed macaques are an edge species (Gumert *et al.* 2009) and tend to live at forest borders in a wide range of habitats. Besides that, they can easily and quickly adapt to the environment. When they inhabit disturbed areas near human settlements, long-tailed macaques quickly learn to raid gardens or crops and beg for food from humans (Lucas & Corlett 1991). Long-tailed macaques are omnivores whereby their primary food choice consists of fruits compared to other types of food (Berenstain 1986). However, they are opportunistic feeders, meaning they can and will eat a wide variety of animals, plants and other materials.

Nowadays, due to human activities, long-tailed macaques’ diet may easily be exposed to heavy metal elements and their diet might be alternate to another food sources. Furthermore, they are keen to feed on food wastes in garbage bins, instead of consuming plants; this habit will affect their health. On the other hand, long-tailed macaques share important immunological and physiological similarities with humans, particularly in the way they respond to toxic exposure (Lee *et al.* 2012). This makes them potentially valuable as a biological indicator for toxic exposures and predictors of physiologic responses to chemicals in humans (Engel *et al.* 2010). A number of criteria are cited as being important for an organism to be a good biological indicator. These include a relatively large body size, sensitivity to the particular agents, similar physiology to humans with a similar route of exposure, a relatively long life, being non-migratory with a wide distribution in the environment, and having a short latent period between exposure and symptom onset (O’Brien *et al.* 1993). Long-tailed macaques satisfy all these criteria for being a good biological indicator. Based on the previous studies, it seems that there are still few researches done regarding to the heavy metal concentration in long-tailed macaques. This study is the first attempt to investigate heavy metal (lead) concentration in long-tailed macaque hair at the study area. Hence, the present study aims to determine the lead (Pb) concentration in hair samples of long-tailed macaques found inside and outside of Kuala Selangor Nature Park (KSNP).

## MATERIALS AND METHODS

### Study Area

**Figure 1:**
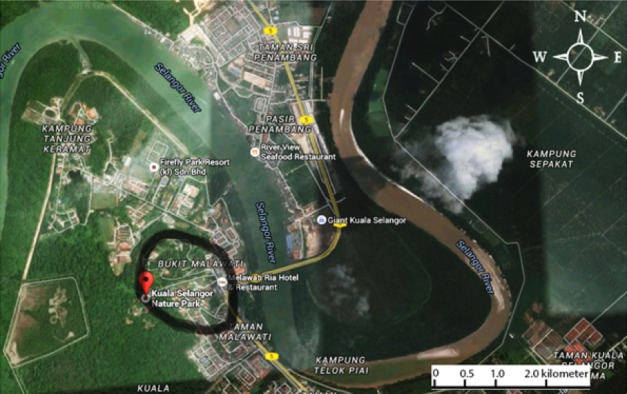
Map of study area (The black circle indicates samples of long-tailed macaque’s hair that were taken). (Source: Google Maps 2018)

This study was conducted at the habitat range of long-tailed macaques in KSNP, Selangor, Malaysia (101° 14.678′E, 03° 20.335′N) and the surrounding area including a small town, Bukit Malawati and a residential area (Fig. 1). Long-tailed macaques outside KSNP were mainly distributed along the road at the entrance of KSNP (Hambali *et al.* 2012); this area is very close to the residential areas. The landform in the study area is horizontal at the road and residential area while there is also a slight slope because the study area is located near Bukit Malawati, Kuala Selangor (Hambali *et al.* 2014a). The ecological niche of long-tailed macaques outside KSNP was overlapping with the human population. The items offered by humans may consist of a variety of fruits such as mangoes, bananas, oranges, langsat, and apples. Besides that, junk food such as nuts, snacks, sweets, and breads are also given by humans (Hambali *et al.* 2014b). Based on the study by Hambali *et al.* (2014b), the group of long-tailed macaques outside KSNP consume food waste from garbage cans while long-tailed macaques inside KSNP mostly consume plant parts, fruits, and insects.

### Hair Sample Collection

Hair samples were collected from six long-tailed macaques trapped at two different locations; three macaques each from inside and outside KSNP (Table 1). The long-tailed macaques were trapped using portable aluminium cage-like trap whereby bananas and several raw chicken eggs were put in the cage as bait to attract them. Once trapped, the long-tailed macaques were hand injected with 5 mg/kg Ketamine HCI to achieve anaesthesia. A ranger from the Department of Wildlife and National Parks (DWNP) helped to make the long-tailed macaques fainted. During anaesthesia, hair specimens were taken manually using latex gloves and surgical scissors, by clipping the hair as close as possible to the skin and extracting, as much as possible, hair strands from each macaque. Moreover, hair samples were cut from three different spots on each macaque’s body. Then, each macaque’s body weight, length and sex were recorded. Hair specimens were placed into sealed and labelled plastic bags and stored at the laboratory refrigerator until processed for analysis.

### Laboratory Analysis

The preparation of sample for lead (Pb) analysis was according to Perkin Elmer’s guidebook. First, each hair sample was treated separately. Hair segments were cut about 5 to 10 mm in length and weighing at least 10 mg. In order to remove the external contamination from the macaque’s hair, the pre-digestion washing technique was conducted to remove only the surface or external contamination without extracting metals from the samples or depositing metals on them. Then, the hair samples were washed in deionised water on a hot plate and then boiled. Next, the sample is transferred to a 100-mL Teflon beaker and digested with a 1:5 mixture of HClO4:HNO3 until a few drops of clear liquid remain. Subsequently, the sample was diluted to 1:50 with deionized water. All analysis was performed at the laboratory of Department of Biology, Faculty of Science, Universiti Putra Malaysia. An Inductively Coupled Plasma Mass Spectrometer (ICP-MS) was used in this study to determine lead concentration.

### Statistical Analysis

Data are presented as mean ± SD (standard deviation). The differences in lead concentration were calculated using Student’s T-test. All statistical analyses were conducted using Microsoft Excel software.

## RESULTS

**Figure 2:**
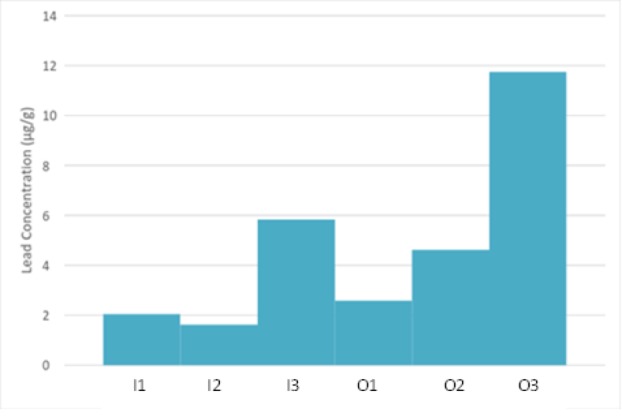
Average concentration of lead in different individuals determined using ICP-MS technique. *I = Inside; O = Outside.

The average mean of Pb, by individual and trapping group, are shown in the Appendix. The results of ICP-MS analysis showed the average lead concentration obtained in μg/g (arithmetic mean = 4.74, SD = 4.28), with a maximum and minimum concentration of 11.74 μg/g and 1.62 μg/g, respectively, and all individual values. The average lead concentration in hair was significantly higher for outside (6.3138±5.2376 μg/g) compared to inside KSNP (3.16±2.25 μg/g). In addition, lead concentrations of individuals varied substantially (Fig. 2).

Based on Figure 2, lead concentration for outside KSNP was significantly higher between individuals with O3, scoring the highest (11.74 μg/g) and O1; scoring the lowest (2.58 μg/g). For inside KSNP, lead concentration was also significantly higher between individuals, with I3 scoring the highest with 5.83 μg/g while I2 scored the lowest with 1.62 μg/g. Moreover, there were statistically significant differences in lead concentrations between males (5.95 ± 2.65 μg/g) and females (2.31 ± 0.67 μg/g) (Table 2).

In addition, the highest lead concentration in the female group was found outside KSNP, which is O1 (2.58 μg/g) while the lowest was inside KSNP, which is I1 (2.04 μg/g). For the male group, the highest lead concentration was found outside KSNP, which is O3 (11.74 μg/g) while the lowest was found inside KSNP, which is I2 (1.62 μg/g).

## DISCUSSION

The data presented here imply that demographic and behavioural variables are associated with lead exposure in long-tailed macaques. Furthermore, an independent effect was seen with trapping locations. A few hypotheses could explain these observations. First, behaviour or ranging pattern may bring some animals into more frequent or intense contact with sources of lead. For example, the macaque is often seen playing in rough and impure areas, hence, coming into more frequent contact with lead-containing soil and dust compared to other animals. Moreover, lead can also be ingested when the animals clean their body. Outside KSNP, there is a source of water from a man-made pond, which the long-tailed macaques use as a place to drink and bathe (Hambali *et al.* 2012).

Since the long-tailed macaque is an opportunistic omnivorous animal (Bonadio 2000), they are likely to feed on many different types of food if their primary food sources are unavailable. This can be attributed to the higher availability of anthropogenic food sources like human garbage and lack of natural food sources in anthropogenic habitats (Sha & Hanya 2013).

In this study, the long-tailed macaque with the highest lead concentration was located outside KSNP. From personal observation, tourists visiting KSNP will feed the monkeys outside KSNP with peanuts, bananas, and long beans purchased from vendors around the area.

Although the real situation of exposure is unknown, ingestion of contaminated plants and water, or inhalation of dust by the monkeys in this area may have caused the observed high exposure to lead. Based on the study by Hambali *et al.* (2014b), long-tailed macaques choose human-sourced food waste in garbage cans available at the area. Besides that, they also find food in the nearby residential area, especially in the trash. In addition, the possibility of the long-tailed macaques looking for remaining food in the trash can cause them to be exposed to polluted materials found in the trash. For example, the long-tailed macaques rummage trash that has lead-based paint cans, food and drink cans, and food waste contaminated with lead.

The Agency for Toxic Substances and Disease Registry (1999) have stated that exposure to lead primarily occurs through inhalation of lead-contaminated dust or aerosols, and ingestion of food, water, and paint contaminated with lead. Common sources of lead include dust containing paint chips or lead released into the atmosphere from industrial or automotive emissions such as leaded gasoline (Goyer 1990).

According to recent reports from Nepal (Engel *et al.* 2010) and Singapore (Schillaci *et al.* 2011), lead concentration in the hair of cynomolgus and rhesus monkeys were 2.51 and 6.00 μg/g, respectively. Meanwhile, lead concentration results in this study were the second highest among these recent reports. The Pb concentration from the present study is lower than the study in Nepal but higher than those reported in Singapore. Furthermore, the Pb concentration reported in China, 0.656 μg/g (Lee *et al*., 2012), is the lowest Pb concentration among those reported in the aforementioned studies. All these studies determined Pb concentration using macaque species as an indicator to environmental exposure. Although this study reported only on lead concentration, further studies on measurement of other heavy metal contaminations, continuous monitoring, adverse effects, and comparison of heavy metal concentration between blood and hair are needed.

## CONCLUSION

The result from this study indicates relatively low lead concentrations in long-tailed macaque hair in KSNP. Nevertheless, the highest lead concentration in long-tailed macaque hair was from outside of the KSNP, which is a tourist attraction area where long-tailed macaques frequently come in contact with the humans. Furthermore, this finding shows that long-tailed macaque could be a good biological indicator for environmental pollutants such as lead. This can be a strategy for monitoring and preparing further remedy before the lead exposure becomes a serious problem to the human population and the environment.

## Figures and Tables

**Table 1:** Information of captured macaques.

Location	No.	Individual	Sex	Body weight (kg)	Length (cm)	Age	Health status
Outside	1	O1	Female	1.445	74	4	Healthy/no injury
	2	O2	Male	2.830	87	4	Healthy/no injury
	3	O3	Male	5.190	101	8	Healthy/no injury

Inside	1	I1	Female	2.830	88	7	Healthy/no injury
	2	I2	Male	2.350	77	2	Healthy/no injury
	3	I3	Male	5.120	114	6	Healthy/no injury

**Table 2:** Comparison of lead concentration according to gender in the hair of long-tailed macaques (μg/g)

Element	Female (*n* = 2)	Male (*n* = 4)	Total (*n* = 6)
Lead (Pb)	2.31 ± 0.67	5.95 ± 2.65	4.74 ± 4.28

## APPENDIX Sample Replication and Results

**Table:**

Replicate	μg/g	Average (μg/g)	SD (μg/g)	Comparison between individual (μg/g)		Comparison between location (μg/g)	
	
				Average	SD	Average	SD
InsideKSNP1Rep1	1.738854	1.730666	0.012289	2.0441705	0.382205157		
InsideKSNP1Rep1	1.73661						
InsideKSNP1Rep1	1.716536						

InsideKSNP1Rep2	2.442561	2.453867	0.011902				
InsideKSNP1Rep2	2.466286						
InsideKSNP1Rep2	2.452754						

InsideKSNP1Rep3	1.951256	1.947978	0.00293				
InsideKSNP1Rep3	1.945615						
InsideKSNP1Rep3	1.947064						
		
InsideKSNP2Rep1	1.446573	1.455414	0.009776	1.618617167	1.83528211	3.1628017	2.25032253
InsideKSNP2Rep1	1.465913						
InsideKSNP2Rep1	1.453756						

InsideKSNP2Rep2	1.670572	1.62718	0.106099				
InsideKSNP2Rep2	1.506263						
InsideKSNP2Rep2	1.704705						

InsideKSNP2Rep3	1.776231	1.773258	0.017095				
InsideKSNP2Rep3	1.788673						
InsideKSNP2Rep3	1.754872						
		
InsideKSNP3Rep1	5.669805	5.666967	0.008497	5.825617444	2.104716403		
InsideKSNP3Rep1	5.673683						
InsideKSNP3Rep1	5.657415						

InsideKSNP3Rep2	7.798856	7.901008	0.088628				
InsideKSNP3Rep2	7.94674						
InsideKSNP3Rep2	7.95743						

InsideKSNP3Rep3	3.92009	3.908877	0.02785				
InsideKSNP3Rep3	3.877168						
InsideKSNP3Rep3	3.929373						
		
OutsideKSNP1Rep1	2.516593	2.512952	0.020729	2.578391333	0.958131551		
OutsideKSNP1Rep1	2.490645						
OutsideKSNP1Rep1	2.53162						

OutsideKSNP1Rep2	2.620148	2.577326	0.039309				
OutsideKSNP1Rep2	2.568954						
OutsideKSNP1Rep2	2.542878						
		
OutsideKSNP1Rep3	2.642288	2.644896	0.014943				
OutsideKSNP1Rep3	2.631428						
OutsideKSNP1Rep3	2.660971						
		
OutsideKSNP2Rep1	4.326808	4.667998	0.348664	4.618700389	0.59050482	6.31387722	5.23763817
OutsideKSNP2Rep1	5.023683						
OutsideKSNP2Rep1	4.653504						

OutsideKSNP2Rep2	5.083629	5.095498	0.01996				
OutsideKSNP2Rep2	5.118543						
OutsideKSNP2Rep2	5.084322						

OutsideKSNP2Rep3	4.051187	4.092605	0.048427				
OutsideKSNP2Rep3	4.08078						
OutsideKSNP2Rep3	4.14585						
		
OutsideKSNP3Rep1	3.160199	3.93861	0.716496	11.74453994	6.068146755		
OutsideKSNP3Rep1	4.085074						
OutsideKSNP3Rep1	4.570557						

OutsideKSNP3Rep2	13.67165	15.80961	3.010117				
OutsideKSNP3Rep2	19.25191						
OutsideKSNP3Rep2	14.50527						

OutsideKSNP3Rep3	14.75494	15.4854	0.733749				
OutsideKSNP3Rep3	15.47885						
OutsideKSNP3Rep3	16.2224						
